# The Timing and Abundance of IL-2Rβ (CD122) Expression Control Thymic *i*NKT Cell Generation and NKT1 Subset Differentiation

**DOI:** 10.3389/fimmu.2021.642856

**Published:** 2021-05-14

**Authors:** Hee Yeun Won, Hye Kyung Kim, Assiatu Crossman, Parirokh Awasthi, Ronald E. Gress, Jung-Hyun Park

**Affiliations:** ^1^ Experimental Immunology Branch, Center for Cancer Research, National Cancer Institute, National Institutes of Health, Bethesda, MD, United States; ^2^ Experimental Transplantation and Immunotherapy Branch, Center for Cancer Research, National Cancer Institute, National Institutes of Health, Bethesda, MD, United States; ^3^ Laboratory Animal Sciences Program, Leidos Biomedical Research, Inc., Frederick National Laboratory for Cancer Research, Frederick, MD, United States

**Keywords:** cytokines, IL-2 (interleukin-2), IL-15, Tbet, thymocytes

## Abstract

Invariant NKT (*i*NKT) cells are thymus-generated innate-like T cells, comprised of three distinct subsets with divergent effector functions. The molecular mechanism that drives the lineage trifurcation of immature *i*NKT cells into the NKT1, NKT2, and NKT17 subsets remains a controversial issue that remains to be resolved. Because cytokine receptor signaling is necessary for *i*NKT cell generation, cytokines are proposed to contribute to *i*NKT subset differentiation also. However, the precise roles and requirements of cytokines in these processes are not fully understood. Here, we show that IL-2Rβ, a nonredundant component of the IL-15 receptor complex, plays a critical role in both the development and differentiation of thymic *i*NKT cells. While the induction of IL-2Rβ expression on postselection thymocytes is necessary to drive the generation of *i*NKT cells, surprisingly, premature IL-2Rβ expression on immature *i*NKT cells was detrimental to their development. Moreover, while IL-2Rβ is necessary for NKT1 generation, paradoxically, we found that the increased abundance of IL-2Rβ suppressed NKT1 generation without affecting NKT2 and NKT17 cell differentiation. Thus, the timing and abundance of IL-2Rβ expression control *i*NKT lineage fate and development, thereby establishing cytokine receptor expression as a critical regulator of thymic *i*NKT cell differentiation.

## Introduction

Cytokines of the common γ-chain (γc) family play critical roles in the generation and differentiation of T cells in the thymus (1–3). Importantly, γc cytokines not only provide prosurvival signals and metabolic cues, but they also specify the lineage commitment of thymocytes and equip developing T cells with effector function (1). Along these lines, a requirement for intrathymic interleukin-7 (IL-7) in T cell development is well established, as documented in its role to control the expansion of immature thymocytes and to determine CD8 cytotoxic T cell lineage fate (2, 4). IL-4, on the other hand, drives the differentiation of innate-like CD8 T cells (5), whereas IL-2 signaling is critical for the generation of Foxp3^+^ T regulatory (Treg) cells (6). Collectively, γc cytokine signaling is necessary for the development of αβ T cells; however, the identity of the required cytokine differs depending on the individual lineages or subsets of T cells that are generated.

Cytokine signaling is also important for the generation of invariant NKT (*i*NKT) cells, a population of innate-like αβ T cells that are produced in the thymus (7, 8). *i*NKT cells are potent immunoregulatory cells that produce pro-inflammatory cytokines, but they differ from conventional αβ T cells because they acquire effector function before antigen encounter and activation (7, 8). Depending on the cytokines they produce and the transcription factors they express, *i*NKT cells can be categorized into three distinct subsets (9, 10). *i*NKT cells that are T-bet^+^ and produce IFNγ correspond to NKT1 cells. IL-17-producing RORγt^+^ *i*NKT cells, on the other hand, are NKT17 cells, and IL-4-producing PLZF^hi^ *i*NKT cells are NKT2 cells (9, 10). The molecular mechanisms that drive *i*NKT subset differentiation are not fully mapped. However, cytokines have been implicated in determining *i*NKT subset specification. Thus, IL-15 promotes NKT1 cell development (11), TGF-β induces NKT17 cell generation (12), and IL-25 produced by thymic tuft cells possibly drives the differentiation of NKT2 cells (13). Consequently, cytokines can specify the differentiation of *i*NKT cells in the thymus. However, it remains unclear why some *i*NKT precursors would respond to IL-15 and become NKT1 cells, whereas other precursors are refractory to IL-15 and choose other lineage fates. This problem is further compounded by all *i*NKT cells expressing the same semi-invariant TCR so that differences in the TCR repertoire are unlikely to play a significant role in subset differentiation. Instead, we consider it likely that differences in the timing or abundance of cytokine receptors could determine the responsiveness to subset-specifying cytokines.

Among *i*NKT cells, the NKT1 subset is of particular interest because it comprises most of the thymic *i*NKT cells in C57BL/6 mice. NKT1 cells are also important because they produce copious amounts of IFNγ, which affects T cell lineage differentiation and promotes the terminal maturation of thymic epithelial cells (14, 15). NKT1 cells require IL-15 for their generation, whereby intrathymic IL-15 is primarily provided by medullary thymic epithelial cells (16, 17). IL-15 is a γc family cytokine that signals through γc and IL-2Rβ (3). Consistent with its requirement for IL-15 signaling, IL-2Rβ deficiency was previously shown to dramatically impair the generation of *i*NKT cells in the thymus (18, 19). However, it remains unclear whether the lack of IL-2Rβ would specifically impede the generation of NKT1 cells or the differentiation of all thymic *i*NKT subsets. Moreover, if IL-2Rβ is *necessary* for NKT1 cell differentiation, we wished to assess whether IL-2Rβ would be *sufficient* to drive NKT1 cell generation. If such were the case, we expected that the forced expression of IL-2Rβ would impose the NKT1 subset fate onto all developing *i*NKT cells, resulting in a heavily skewed *i*NKT subset composition where NKT1 cells would be overrepresented and NKT2 and NKT17 cells would be underrepresented. To this end, we examined the generation and differentiation of *i*NKT cells in IL-2Rβ^Tg^ mice in both the C57BL/6 and BALB/c backgrounds, and further generated mice with conditional deletion of IL-2Rβ upon *i*NKT lineage commitment. Here, we show that not only the expression itself but also the carefully curated timing of IL-2Rβ induction is important for the generation and subset differentiation of *i*NKT cells. Collectively, these results unveil previously unappreciated roles and requirements of the cytokine receptor IL-2Rβ in controlling thymic *i*NKT cell development.

## Materials and Methods

### Mice

C57BL/6NCrl (C57BL/6) and BALB/cAnNCrl (BALB/c) mice were obtained from the Charles River Laboratories (Frederick, MD). IL-2Rβ^Tg^ mice were generated in house as previously described (20). IL-2Rβ-deficient mice (*Il2rb* ^–/–^) and IL-2Rβ-floxed mice (*Il2rb* ^fl/fl^) were obtained from the Jackson Laboratories (21, 22). PLZF^Cre^ transgenic mice were kindly provided by Dr. D. Sant’Angelo (Rutgers University, NJ) (23). CA-STAT5^Tg^ mice were a kind gift from by Dr. M. A. Farrar (U. Minnesota, MN) (24). T-bet-ZsGreen reporter transgenic mice (TBGR^Tg^) on C57BL/6 background were a kind gift from Dr. Jinfang Zhu (NIAID, NIH) (25), and these mice were backcrossed to BALB/cAnNCrl for at least 6 generations before analysis. All experimental mice were analyzed between 6 and 12 weeks of age, except for *Il2rb* ^–/–^ and their littermate controls, which were analyzed between 4 and 6 weeks of age. All mice were cared for in accordance with NIH guidelines. Animal experiments were approved by the NCI Animal Care and Use Committee.

### Flow Cytometry

Single-cell suspensions were prepared from the indicated tissues and stained with fluorescence-conjugated antibodies as previously described (26). The data were acquired using LSR Fortessa or LSRII flow cytometers (BD Biosciences) and were analyzed using software platforms developed by the EIB Flow Cytometry Facility, CCR, NCI, NIH. Live cells were gated by forward scatter exclusion of dead cells stained with propidium iodide. The following antibodies were used for staining: HSA (M1/69), T-bet (4B10), Foxp3 (FJK-16s), RORγt (AKFJS-9) and isotype control antibodies, all from eBioscience; CD4 (GK1.5 and RM4.5), CD8α (53–6–7), CD69 (H1.2F3), TCRβ (H57-597) and IL2Rβ (TM-β1) from BD Biosciences; CD44 (IM7), NK1.1 (PK136), IL2Rα (PC61), CCR7 (4B12) and PLZF (9E12) from BioLegend. Fluorochrome-conjugated CD1d tetramers loaded with PBS-57 (CD1dTet) and unloaded controls were obtained from the NIH tetramer facility (Emory University, Atlanta, GA). Intracellular Foxp3, PLZF, RORγt, and T-bet proteins were detected using a Foxp3 staining kit according to the manufacturer’s instructions (eBioscience Thermo Fisher).

### *i*NKT Subset Identification by Transcription Factors

To identify the subset composition of *i*NKT cells, total thymocytes were first stained with PBS-57-loaded mouse CD1d tetramers, fixed, permeabilized and then stained for nuclear transcription factors. Briefly, thymocytes were stained with fluorochrome-conjugated CD1d tetramers in FACS buffer (0.5% BSA, 0.1% sodium azide in Ca^2+^ and Mg^2+^-free HBSS) for 20 minutes at 4°C. Without removing the tetramer reagents, the cells were incubated for an additional 30 min at 4°C with antibodies for surface markers. Excess reagents were then washed out with FACS buffer, and the cells were resuspended in a 1:3 mixture of concentrate/diluent working solution of the Foxp3 Transcription Factor Staining Buffer kit (eBioscience Thermo Fisher), followed by incubation at room temperature for 20 minutes. Next, the cells were washed twice with 1× permeabilization buffer (eBioscience Thermo Fisher) before adding antibodies for transcription factors, specifically antibodies against PLZF, RORγt and T-bet. The cells were incubated at room temperature for 1 hour before washing out excess reagents with FACS buffer and analysis by flow cytometry.

### Alzet Osmotic Pump Installation

Recombinant mouse IL-15 (Peprotech) dissolved in PBS was administered into IL-2Rβ^Tg^ BALB/c mice using Alzet osmotic pumps (Durect) following the manufacturer’s instruction. The pumps (model 1002) were set to release IL-15 at a rate of 3 µg of recombinant IL-15 per 24 hours.

### Statistical Analysis

The data were shown as the means ± SEM. Statistical significance was determined by unpaired two-tailed Student’s *t*-test. *P* values of less than 0.05 were considered significant. *P < 0.05, **P < 0.01, ***P <0.001. All statistical analysis was performed using GraphPad Prism 7 software (GraphPad software).

## Results

### IL-2Rβ Expression Is Induced Upon Thymic *i*NKT Cell Differentiation

**Figure 1:**
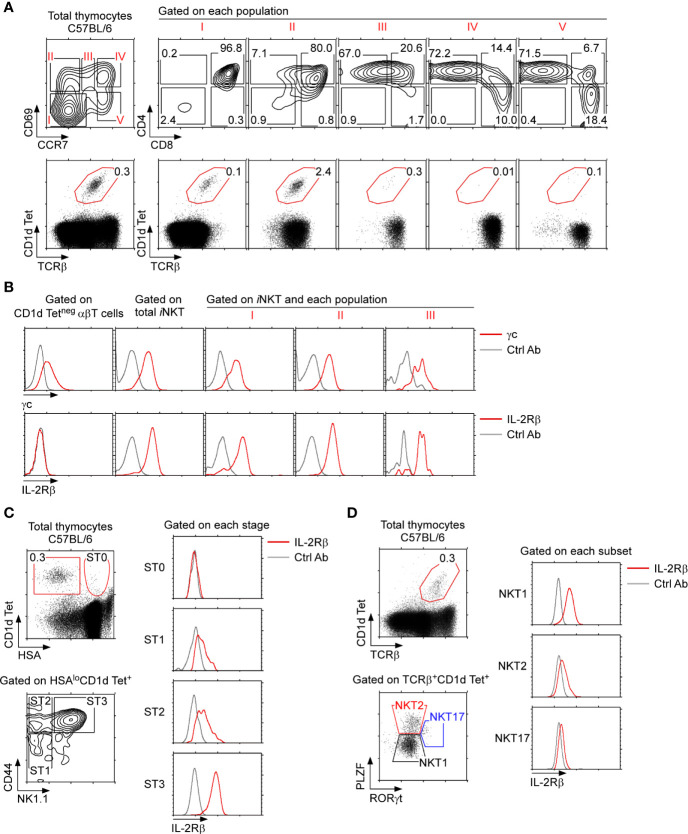
IL-2Rβ expression on thymic *i*NKT cells **(A)** C57BL/6 thymocytes were assessed for surface CD69 and CCR7 staining, which visualizes 5 distinct stages of differentiation (*i.e.*, stages I–V), whereby thymocytes undergoing positive selection correspond to population III (top). *i*NKT cells were identified by PBS57-loaded CD1d tetramer (CD1dTet) staining among the 5 stages defined by CD69 and CCR7 (bottom). The results are representative of 5 independent experiments with a total of 5 WT C57BL/6 mice. **(B)** Surface cytokine receptor expression on thymic *i*NKT cell populations. *i*NKT cells in populations I, II, and III were assessed for γc and IL-2Rβ expression (red lines). Isotype control antibody staining are shown as gray lines. The results are representative of 3 independent experiments. **(C)** IL-2Rβ expression during *i*NKT cell differentiation. Thymic *i*NKT cells were divided into immature ST0 and mature stage 1–3 cells based on HSA expression (left, top). The abundance of IL-2Rβ was then assessed on ST0 (HSA^hi^CD44^–^), ST1 (CD44^–^NK1.1^–^), ST2 (CD44^+^NK1.1^–^), and ST3 (CD44^+^NK1.1^+^) *i*NKT cells (left, bottom). Histograms show surface IL-2Rβ expression for each subset (red line). Isotype control antibody staining are shown as gray lines. The results are representative of 5 independent experiments. **(D)** IL-2Rβ expression in thymic *i*NKT subsets. *i*NKT cells were assessed for intracellular PLZF and RORγt expression to identify NKT1, NKT2, and NK17 cells (left). Each *i*NKT subset was assessed for IL-2Rβ expression (right, red lines). Isotype control antibody staining are shown as gray lines. The results are representative of 3 independent experiments.

To understand the role of IL-2Rβ in thymic *i*NKT cell generation, we first examined IL-2Rβ expression on *i*NKT cells in the context of αβ T cell development in the thymus. The process of thymocyte positive selection and maturation can be visualized using the combination of the two surface markers CD69 and CCR7 which identifies 5 distinct developmental stages, progressing from stage I to stage V (**Figure 1A**, top) (27). TCR engagement of immature thymocytes (stage I) induces the expression CD69 (stage II) followed by the upregulation of the chemokine receptor CCR7, so that CD69^+^CCR7^int^ cells (stage III) correspond to thymocytes undergoing positive selection and lineage commitment. Stage IV and V cells, on the other hand, correspond to postselection thymocytes. We found that *i*NKT cells, as identified by PBS-57-loaded CD1d tetramers (CD1dTet^+^), arise as early as in stage I, and that they were then prominent in stage II (**Figure 1A**, bottom). Importantly, stage I *i*NKT cells expressed both the cytokine receptor γc and IL-2Rβ (**Figure 1B** and **Supplementary Figure 1**), indicating that the induction of IL-2Rβ is one of the earliest events in *i*NKT cell development. Indeed, such kinetics of IL-2Rβ expression differed substantially from that of CD1dTet-negative conventional αβ T cells, which remained mostly absent for IL-2Rβ expression throughout their development (**Figure 1B** and **Supplementary Figure 2**).

To further align IL-2Rβ expression with the selection and maturation of *i*NKT cells, we next assessed surface IL-2Rβ expression in developmental stages of thymic *i*NKT cells. CD44-negative HSA^hi^ thymocytes that bind PBS-57-loaded CD1d tetramers correspond to preselection *i*NKT cells, and they are usually referred to as stage 0 (ST0) *i*NKT cells (28). IL-2Rβ was mostly absent on ST0 *i*NKT cells but was induced following differentiation into HSA^lo^ mature *i*NKT cells (**Figure 1C** and **Supplementary Figure 1B**). Thymic differentiation of HSA^lo^ *i*NKT cells proceeds along a well-characterized pathway that is marked by the expression of CD44 and NK1.1, whereby CD44^–^NK1.1^–^ cells correspond to stage 1 (ST1), followed by CD44^+^NK1.1^–^ cells that are stage 2 (ST2), and terminally differentiate into CD44^+^NK1.1^+^ stage 3 cells (ST3) (28). Interestingly, IL-2Rβ was expressed at low levels on ST1 and ST2 cells but highly expressed on ST3 *i*NKT cells (**Figure 1C** and **Supplementary Figure 1B**). Thus, all mature thymic *i*NKT cells express IL-2Rβ to a certain degree, but the amount of surface IL-2Rβ proteins substantially increases with maturation.

Mature *i*NKT cells are also categorized into 3 distinct subsets, namely NKT1, NKT2, and NKT17 cells, based on their transcription factor expression profiles (10). Differential expression of the transcription factors PLZF *versus* RORγt can visualize these three subsets, which we utilized to assess IL-2Rβ expression on individual thymic *i*NKT subsets (**Figure 1D**). Consistent with previous observations (10), IL-2Rβ was highly induced on NKT1 cells and minimally expressed on PLZF^hi^RORγt^neg^ NKT2 and PLZF^int^RORγt^+^ NKT17 cells (**Figure 1D**). Altogether, these results demonstrated that IL-2Rβ is highly upregulated on terminally differentiated ST3 *i*NKT cells and that such IL-2Rβ^+^ *i*NKT cells correspond to NKT1 cells.

### Conditional Deletion of IL-2Rβ in PLZF^+^ Thymocytes

**Figure 2:**
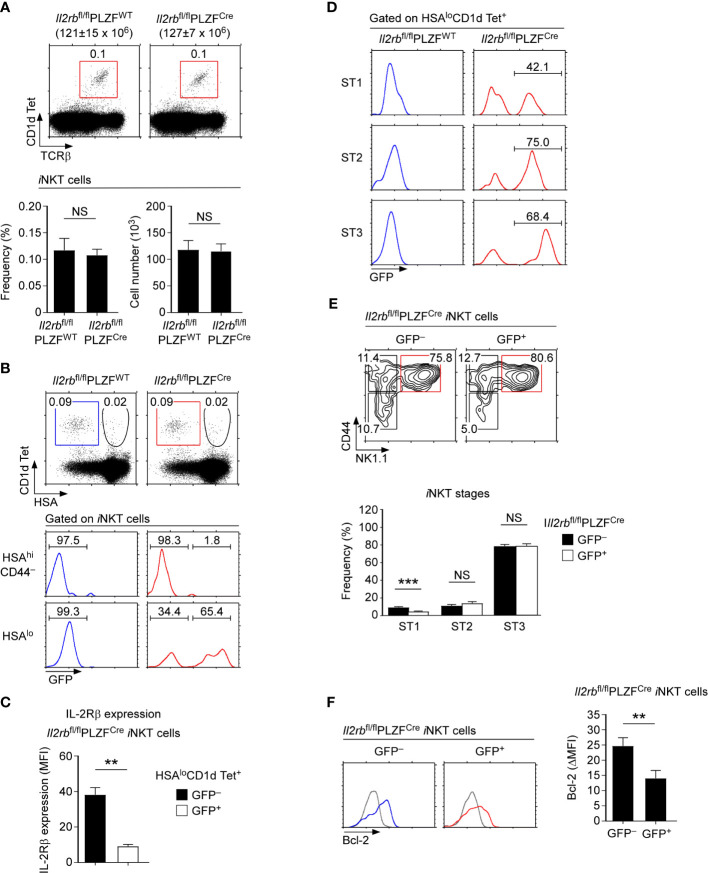
*i*NKT cell generation and differentiation in IL-2Rβ-floxed PLZF-Cre mice **(A)** Frequency and number of thymic *i*NKT cells in *Il2rb* ^fl/fl^PLZF^Cre^ and *Il2rb* ^fl/fl^ littermate control mice. The dot plots show thymic *i*NKT cells as identified by CD1dTet *versus* TCRβ staining (top). The bar graphs show the frequencies and numbers of *i*NKT cells from the indicated mice (bottom). The dot plot is representative, and the bar graphs are a summary of 4 independent experiments with a total of 11 *Il2rb* ^fl/fl^PLZF^Cre^ and 5 *Il2rb* ^fl/fl^ littermate control mice. **(B)** IL-2Rβ-deletion efficiency in *Il2rb* ^fl/fl^PLZF^Cre^ *i*NKT cells. The dot plots show thymic *i*NKT cells of the indicated mice CD1dTet *versus* HSA staining. HSA^hi^CD44^–^ cells correspond to ST0 immature *i*NKT cells while HSA^lo^ cells are mature ST1-3 *i*NKT cells. The histograms show GFP expression among the indicated population of thymic *i*NKT cells. **(C)** IL-2Rβ expression in GFP^+^ and GFP-negative mature *i*NKT cells of *Il2rb* ^fl/fl^PLZF^Cre^ *i*NKT mice. Bar graphs show the summary of analyzing 6 *Il2rb* ^fl/fl^PLZF^Cre^ mice. **(D)** GFP expression in ST1, ST2, and ST3 *i*NKT cells of *Il2rb* ^fl/fl^PLZF^Cre^ and littermate control thymocytes. The data are representative of 4 independent experiments. **(E)** Thymic *i*NKT cell differentiation in *Il2rb* ^fl/fl^PLZF^Cre^ mice. *i*NKT cells were stained for CD44 and NK1.1 to identify ST1, ST2, and ST3 cells as shown in the contour plots (top). The bar graph shows the frequencies of ST1, ST2, and ST3 cells in GFP^+^ and GFP^–^ thymic *i*NKT cells of *Il2rb* ^fl/fl^PLZF^Cre^ mice (bottom). Results show the summary of 6 independent experiments with a total of 14 *Il2rb* ^fl/fl^PLZF^Cre^ mice. **(F)** Bcl-2 expression in GFP^+^ and GFP^–^ *i*NKT cells of *Il2rb* ^fl/fl^PLZF^Cre^ thymocytes. Histograms are representative (left), and the bar graph (right) shows the summary of 3 independent experiments with a total of 8 *Il2rb* ^fl/fl^PLZF^Cre^ mice. **P < 0.01; ***P < 0.001. NS, Not Significant.

Having established that IL-2Rβ expression is *associated* with *i*NKT cell maturation, we next wished to examine whether IL-2Rβ would be also *required* for the development and differentiation of *i*NKT cells. To this end, we aimed to set up an experimental system where IL-2Rβ would be selectively deleted in *i*NKT cells. The zinc finger transcription factor PLZF is expressed in *i*NKT cells but absent in conventional T cells (29, 30). Thus, we bred PLZF-Cre transgenic mice (PLZF^Cre^) with *Il2rb* ^fl/fl^ mice (21, 23) to generate *Il2rb* ^fl/fl^PLZF^Cre^ mice and to specifically delete IL-2Rβ in *i*NKT cells. In such *Il2rb* ^fl/fl^PLZF^Cre^ mice, the thymic development of conventional T cells was unaffected (**Supplementary Figure 3A**). Both TCRβ expression and the CD4 *versus* CD8 profiles of *Il2rb* ^fl/fl^PLZF^Cre^ thymocytes remained comparable to those of littermate *Il2rb* ^fl/fl^PLZF^WT^ thymocytes (**Supplementary Figure 3A**). Contrary to our expectation, however, the frequency and number of thymic *i*NKT cells also remained unaltered in *Il2rb* ^fl/fl^PLZF^Cre^ mice (**Figure 2A**).

To understand these results, we considered two possibilities. Firstly, IL-2Rβ expression in the thymus could be dispensable for *i*NKT cell generation. Alternatively, the PLZF-Cre-mediated deletion of IL-2Rβ could be incomplete. In the latter case, the variegated expression of the PLZF-Cre or inefficient excision of the floxed allele could result in the retention of IL-2Rβ expression. To discriminate these possibilities, it would be necessary to monitor the deletion efficiency of IL-2Rβ in *Il2rb* ^fl/fl^PLZF^Cre^ mice. Notably, the *Il2rb*-floxed allele (*Il2rb* ^fl^) is engineered to induce the expression of Green Fluorescent Proteins (GFP) upon IL-2Rβ deletion (21), permitting the identification of *i*NKT cells that have deleted IL-2Rβ. Employing this feature, we next analyzed the efficiency of IL-2Rβ deletion by assessing GFP expression. Among HSA^hi^ immature *i*NKT cells (ST0), GFP was only found in a very small fraction of *i*NKT cells (**Figure 2B**, top histogram). Among HSA^lo^ *i*NKT cells, however, a large fraction of cells turned out to be GFP^+^, reporting the successful deletion of the *Il2rb* ^fl^ allele in mature *i*NKT cells (**Figure 2B**, bottom histogram, and **Supplementary Figure 3B**). Assessing the surface IL-2Rβ expression on GFP^+^ and GFP-negative mature *i*NKT cells showed a substantial loss of surface IL-2Rβ on GFP^+^ cells (**Figure 2C** and **Supplementary Figure 3C**). Thus, GFP^+^ cells correspond to *i*NKT cells that have terminated IL-2Rβ expression. Importantly, the frequencies of such GFP^+^ *i*NKT cells increased upon their further differentiation into ST1, ST2, and ST3 *i*NKT cells (**Figure 2D**). These results suggested that PLZF^Cre^- mediated deletion of IL-2Rβ is initiated in ST0 cells but not accomplished until later stages of *i*NKT cell development. Because of this delayed deletion, significant amounts of surface IL-2Rβ remained on GFP^+^ *i*NKT cells which could provide residual IL-2R signaling (**Figure 2C**). Along these lines, we noted that a significant fraction of HSA^lo^ mature *i*NKT cells remained GFP-negative and fully expressed IL-2Rβ (**Figure 2B** bottom). Such inefficient deletion could explain why we did not find significant differences in the frequency and number of thymic *i*NKT cells between *Il2rb* ^fl/fl^PLZF^Cre^ and littermate control mice (**Figure 2A**). We also did not find any significant changes in the ST1-ST3 distribution of GFP^+^ *i*NKT cells which expressed dramatically lower amounts of IL-2Rβ compared to GFP-negative *i*NKT cells (**Figure 2E**). We also did not find differences in the ST1-3 distribution of *Il2rb* ^fl/fl^PLZF^Cre^ mice compared to *Il2rb* ^fl/fl^PLZF^WT^ littermates that express normal amounts of IL-2Rβ (**Supplementary Figure 3D**). Along these lines, the thymic *i*NKT subset composition and the frequency of T-bet^+^ *i*NKT cells which correspond to ST3 cells were virtually unaltered in *Il2rb* ^fl/fl^PLZF^Cre^ mice (**Supplementary Figures 4A, B**). On the other hand, the protein abundance of Bcl-2, a direct downstream molecule of IL-2Rβ signaling (31), was markedly diminished (**Figure 2F**), indicating that the lack of IL-2Rβ had physiological consequences for *i*NKT cells. Nonetheless, the survival of GFP^+^ thymic *i*NKT cells remained unaffected, because Bcl-xL and not Bcl-2 provides pro-survival signals for *i*NKT cells (11). In agreement the frequency and number as well as the subset composition of iNKT cells in the spleen did not differ between *Il2rb* ^fl/fl^PLZF^Cre^ and *Il2rb* ^fl/fl^PLZF^WT^ mice (**Supplementary Figure 4C**). Collectively, the undisturbed differentiation of GFP^+^ *i*NKT cells in *Il2rb* ^fl/fl^PLZF^Cre^ mice suggested that the late-stage deletion of IL-2Rβ is not detrimental for the ST1 to ST3 *i*NKT cell maturation.

### IL-2Rβ Is Critical for the Generation of Thymic *i*NKT Cells

**Figure 3:**
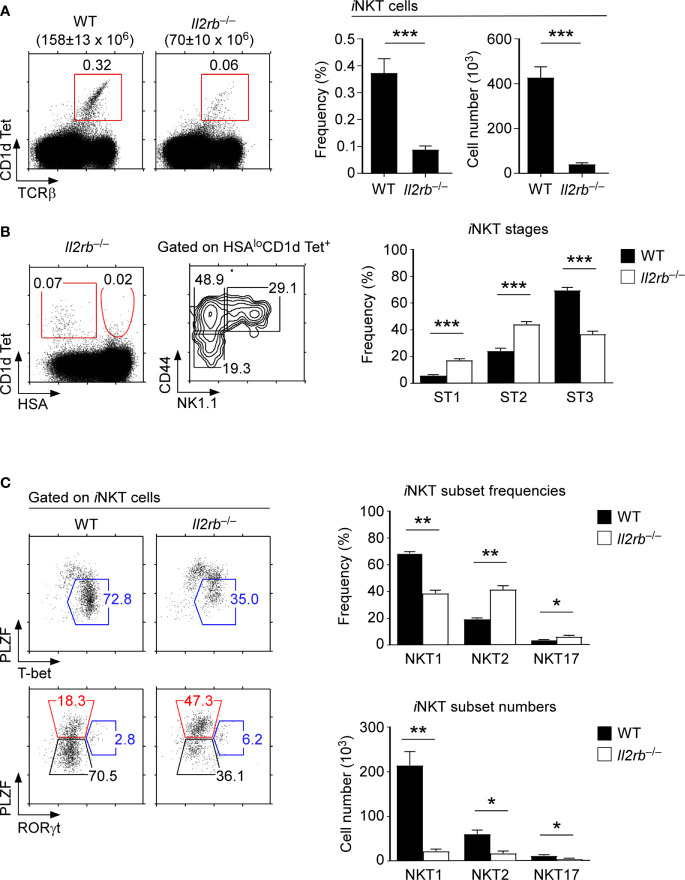
An IL-2Rβ requirement for thymic *i*NKT cell differentiation **(A)** Frequency and number of thymic *i*NKT cells in *Il2rb* ^–/–^ and WT littermate mice. Dot plots show thymic *i*NKT cells as identified by CD1dTet and TCRβ staining. Total thymocyte numbers are shown above the dot plots as the means ± SEM (left). Bar graphs show the frequencies and numbers of *i*NKT cells from the indicated mice (right). The dot plot is representative, and the bar graphs are a summary of 4 independent experiments with a total of 13 *Il2rb* ^–/–^ and 7 WT littermate mice. **(B)** Thymic *i*NKT cell differentiation in *Il2rb* ^–/–^ mice. HSA^lo^ mature *i*NKT cells were stained for CD44 and NK1.1 to identify ST1, ST2, and ST3 cells in the indicated mice (left). The bar graph shows the frequencies of ST1, ST2, and ST3 cells in *Il2rb* ^–/–^ and WT littermate mice (right). The dot and contour plots are representative, and the bar graph shows the summary of 4 independent experiments. **(C)** Dot plots show the thymic *i*NKT subset composition in *Il2rb* ^–/–^ and WT littermate mice (left). The bar graph on top shows the frequency of thymic *i*NKT subsets as summary of 5 independent experiments with a total of 14 *Il2rb* ^–/–^ and 8 WT littermate mice. The bottom bar graph shows the cell numbers of thymic *i*NKT subsets as the summary of 4 independent experiments with a total of 9 *Il2rb* ^–/–^ and 4 WT littermate control mice. *P < 0.05; **P < 0.01; ***P < 0.001.

To further delineate the role of IL-2Rβ in the generation and differentiation of *i*NKT cells, we next analyzed the development and subset composition of thymic *i*NKT cells in germline IL-2Rβ-deficient (*Il2rb* ^–/–^) mice (22). In *Il2rb* ^–/–^ mice, all thymocytes - including ST0 immature *i*NKT cells - are devoid of IL-2Rβ. Thus, an IL-2Rβ requirement for *i*NKT cells can be assessed starting at the earliest precursor stages of *i*NKT cell development. Notably, *Il2rb* ^–/–^ mice are autoimmune because of the impaired generation of immunosuppressive Foxp3^+^ Treg cells that require IL-2 signaling for their maturation (32, 33). Consequently, we analyzed *Il2rb* ^–/–^ mice before 6 weeks of age to avoid the potential skewing of *i*NKT cell differentiation due to autoimmunity. We confirmed that the thymus of *Il2rb* ^–/–^ mice at that age did not show an overtly activated phenotype, and that T cell development remained comparable to that of WT littermate mice (**Supplementary Figure 5A**). We further confirmed the lack of *in vivo* IL-2Rβ signaling in *Il2rb* ^–/–^ mice by their absence of mature CD25^+^Foxp3^+^ Treg cells which require IL-2R signaling for their generation (**Supplementary Figure 5B**) (32, 33). Consistent with previous reports (18, 19), we confirmed a dramatic reduction in the frequency and number of thymic *i*NKT cells of *Il2rb* ^–/–^ mice compared with those of WT littermate mice (**Figure 3A**). Notably, the loss was specific for *i*NKT cells and did not affect conventional αβ T cells, resulting in a dramatically decreased ratio of *i*NKT cells over αβ T cells (**Supplementary Figure 5C**).

Most thymic *i*NKT cells in C57BL/6 background mice correspond to ST3 cells. Therefore, we expected that IL-2Rβ-deficiency would selectively affect the differentiation of ST3 *i*NKT cells. Indeed, *Il2rb* ^–/–^ thymic *i*NKT cells were developmentally arrested, resulting in the accumulation of ST1/ST2 *i*NKT cells and in a substantial decrease in ST3 *i*NKT cells (**Figure 3B**). We further found that the fraction of NKT1 cells was significantly diminished among *Il2rb* ^–/–^ *i*NKT cells, which in turn resulted in the relative overrepresentation of NKT2 and NKT17 cells (**Figure 3C**). The *i*NKT cell number for each subset, however, were significantly reduced (**Figure 3C**), which was consistent with the dramatic decrease in total *i*NKT cell numbers in *Il2rb* ^–/–^ mice (**Figure 3A**). Collectively, these results from *Il2rb* ^–/–^ mice documented a major role for the cytokine receptor IL-2Rβ in the generation, but also in the subset differentiation of thymic *i*NKT cells.

### Forced Expression of IL-2Rβ Suppresses *i*NKT Cell Development in the Thymus

**Figure 4:**
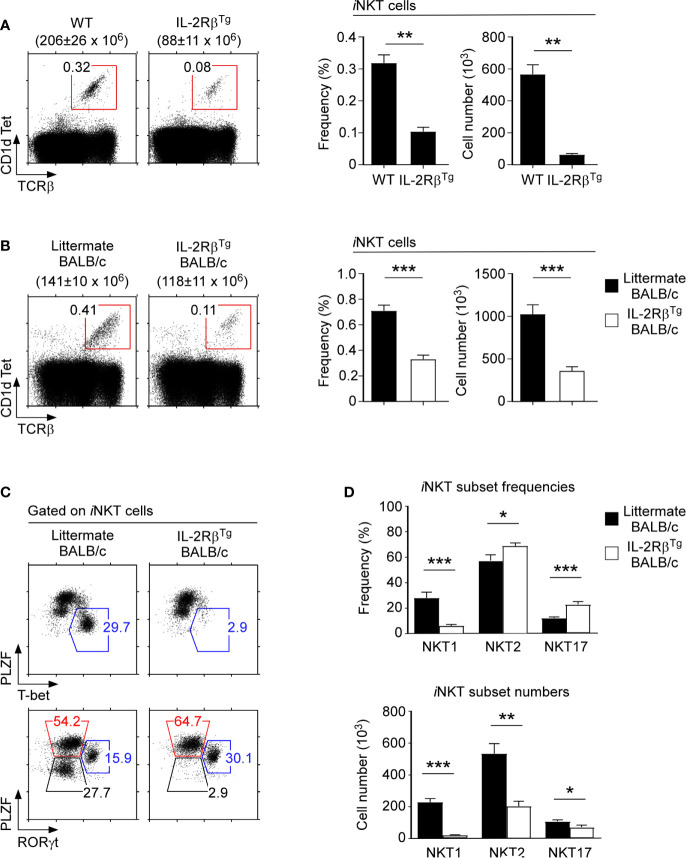
*i*NKT cell differentiation in IL-2Rβ transgenic mice **(A)** Frequency and number of thymic *i*NKT cells in IL-2Rβ^Tg^ and littermate C57BL/6 mice. The dot plots show thymic *i*NKT cells as identified by CD1dTet *versus* TCRβ staining (left). The total thymocyte numbers are shown above the dot plots as the means ± SEM. The bar graphs show the frequencies and numbers of *i*NKT cells from the indicated mice (right). The dot plot is representative, and the bar graphs are a summary of 4 independent experiments with a total of 7 IL-2Rβ^Tg^ and 6 littermate C57BL/6 mice. **(B)** Frequency and number of thymic *i*NKT cells in IL-2Rβ^Tg^ BALB/c and littermate WT mice. The dot plots show thymic *i*NKT cells as identified by CD1dTet *versus* TCRβ staining (left). The total thymocyte numbers are shown above the dot plots as the means ± SEM. The bar graphs show the frequencies and numbers of *i*NKT cells from the indicated mice (right). The dot plot is representative, and the bar graphs are a summary of 3 independent experiments with a total of 7 IL-2Rβ^Tg^ and 8 littermate WT mice. **(C)** The dot plots show the thymic *i*NKT subset composition in IL-2Rβ^Tg^ BALB/c and WT littermate mice based on PLZF *versus* T-bet (top) and PLZF *versus* RORγt analysis (bottom). Results are representative of 4 independent experiments. **(D)** The bar graphs show the summary of thymic *i*NKT subset distribution for the indicated mice. Subset frequencies are the summary of 5 independent experiments with a total of 10 IL-2Rβ^Tg^ and 10 littermate WT BALB/c mice (top). Cell numbers of each *i*NKT subset are the summary of 4 independent experiments with a total of 8 IL-2Rβ^Tg^ and 7 littermate WT BALB/c control mice (bottom). *P < 0.05; **P < 0.01; ***P < 0.001.

Because IL-2Rβ is important for NKT1 cell generation (**Figure 3C**), we next asked whether IL-2Rβ would be also sufficient to impose NKT1 lineage fate during *i*NKT cell development. To this end, we assessed *i*NKT cell development in IL-2Rβ-transgenic mice (IL-2Rβ^Tg^) that overexpress mouse IL-2Rβ under the control of human *CD2* promoter/enhancer elements (20, 34). In these animals, the transgenic IL-2Rβ is prematurely expressed on preselection thymocytes (**Supplementary Figure 6A**) and further overexpressed on postselection mature T cells (**Supplementary Figure 6A**). Consequently, the IL-2Rβ is abundantly expressed on both immature and mature thymocytes of IL-2Rβ^Tg^ mice. Among *i*NKT cells, immature HSA^hi^CD44^–^ *i*NKT cells (ST0) normally do not express IL-2Rβ (**Supplementary Figure 6B**). However, the ST0 *i*NKT cells in IL-2Rβ^Tg^ mice displayed substantially increased abundance of IL-2Rβ (**Supplementary Figure 6B**). Importantly, such premature expression of IL-2Rβ dramatically impaired the generation of *i*NKT cells, so that the frequency and number of thymic *i*NKT cells in IL-2Rβ^Tg^ mice were markedly reduced (**Figure 4A**). These results suggested that IL-2 receptor signaling in immature *i*NKT is detrimental to the generation of thymic *i*NKT cells. These findings also indicated that the timing of IL-2Rβ expression during *i*NKT cell development needs to be carefully controlled, presumably to protect immature *i*NKT cells from premature IL-2 or IL-15 signaling.

### Forced Expression of IL-2Rβ Suppresses the Generation of NKT1 Cells in BALB/c Mice

Because IL-2Rβ is associated with NKT1 cell differentiation, we next aimed to interrogate if premature IL-2Rβ expression in immature ST0 *i*NKT cells would affect the development of other *i*NKT subsets. In C57BL/6 mice, most thymic *i*NKT cells correspond to NKT1 cells (10), making it difficult to discern how the IL-2Rβ^Tg^ would affect NKT2 and NKT17 cells which are underrepresented in this mouse strain. BALB/c mice differ from C57BL/6 mice in their thymic *i*NKT subset composition as it is enriched in NKT2 and NKT17 cells and reduced in NKT1 cells compared with that in C57BL/6 mice (10). Thus, we considered BALB/c mice as an appropriate model to assess the effects of premature IL-2Rβ expression on NKT2 and NKT17 cell generation. To this end, we backcrossed the IL-2Rβ^Tg^ onto the BALB/c background and analyzed the development and differentiation of thymic *i*NKT cells in these mice.

As expected, the thymocytes of IL-2Rβ^Tg^ BALB/c mice expressed substantially increased amounts of surface IL-2Rβ compared with the thymocytes of littermate mice (**Supplementary Figure 6C**). Such increase in the IL-2Rβ abundance was also observed in HSA^hi^CD44^–^ (ST0) immature *i*NKT cells (**Supplementary Figure 6D**), but curiously not in HSA^lo^ mature *i*NKT cells (**Supplementary Figure 6D**). These results suggested that the expression of the IL-2Rβ^Tg^ is either silenced in HSA^lo^ *i*NKT cells or that the premature expression of IL-2Rβ in HSA^hi^CD44^–^ (ST0) immature *i*NKT cells is detrimental for *i*NKT cell generation so that only *i*NKT cells that have escaped the transgene expression can mature. In fact, we found that the frequency and number of thymic *i*NKT cells were indeed dramatically reduced in IL-2Rβ^Tg^ BALB/c mice (**Figure 4B**). Thus, consistent with our findings in IL-2Rβ^Tg^ C57BL/6 mice (**Figure 4A**), the forced expression of IL-2Rβ suppresses the generation of thymic *i*NKT cells in BALB/c mice also (**Figure 4B**).

To understand the effects of IL-2Rβ on *i*NKT cell differentiation, we next examined the *i*NKT subset composition in IL-2Rβ^Tg^ BALB/c mice. Surprisingly, NKT1 cells, as identified by T-bet expression, were virtually absent in IL-2Rβ^Tg^ BALB/c thymocytes. Both the frequency and number of T-bet^+^ NKT1 cells were dramatically decreased in these mice (**Figures 4C, D**). Conversely, NKT2 and NKT17 cell frequencies were significantly increased in IL-2Rβ^Tg^ BALB/c mice compared with those of WT littermate controls (**Figures 4C, D**). Thus, transgenic IL-2Rβ selectively inhibited the development of NKT1 cells without suppressing the generation of NKT2 and NKT17 cells.

**Figure 5:**
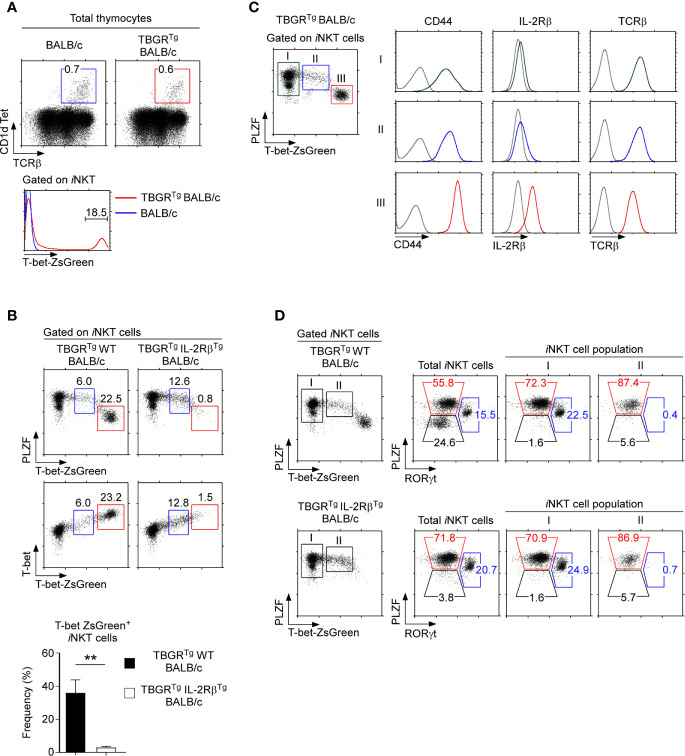
T-bet-ZsGreen reporter expression in *i*NKT cells of IL-2Rβ^Tg^ BALB/c mice **(A)** Thymic *i*NKT cells were identified in BALB/c and TBGR^Tg^ BALB/c mice (top) and assessed for T-bet-ZsGreen reporter expression (bottom). Results are representative of 5 independent experiments. **(B)** T-bet-ZsGreen reporter expression in thymic *i*NKT cells of TBGR^Tg^IL-2Rβ^Tg^ BALB/c and TBGR^Tg^ littermate control mice. Dot plots show either PLZF *versus* T-bet-ZsGreen reporter expression (top) or T-bet protein *versus* T-bet-ZsGreen reporter expression (bottom) in HSA^lo^ thymic *i*NKT cells of TBGR^Tg^ IL-2Rβ^Tg^ BALB/c and TBGR^Tg^ WT littermate mice. Bar graph shows the frequency of T-bet-ZsGreen^+^ *i*NKT cells (red box) of the indicated mice. Results are representative 3 independent experiments with 8 TBGR^Tg^IL-2Rβ^Tg^ BALB/c and 5 TBGR^Tg^ littermate WT BALB/c mice. **(C)** T-bet-ZsGreen reporter expression upon NKT1 cell differentiation. Thymic *i*NKT cells of TBGR^Tg^ BALB/c mice were divided into three distinct populations, *i.e.* I, II, and III, based on the amount of T-bet-ZsGreen expression (dot plot, left). Surface expression of CD44, IL-2Rβ, and TCRβ was then assessed for each *i*NKT population (histograms, right). Results are representative of 2 independent experiments with a total of 6 TBGR^Tg^ BALB/c mice. **(D)** Subset compositions of thymic *i*NKT cells in TBGR^Tg^ BALB/c mice based on ZsGreen-reporter expression. Total *i*NKT cells, T-bet-ZsGreen-negative (population I), and T-bet-ZsGreen-intermediate (population II) *i*NKT cells were identified in TBGR^Tg^ WT BALB/c (top) and TBGR^Tg^IL-2Rβ^Tg^ BALB/c thymocytes (bottom) and examined for their subset composition based on PLZF *versus* RORγt staining. Results are representative of 3 independent experiments with 8 TBGR^Tg^IL-2Rβ^Tg^ BALB/c and 5 TBGR^Tg^ littermate WT BALB/c mice. **P < 0.01.

To further examine how IL-2Rβ^Tg^ would interfere with the induction of T-bet and impair NKT1 cell generation, we employed the T-bet-ZsGreen reporter mouse (TBGR^Tg^) to monitor T-bet transcription in IL-2Rβ^Tg^ and WT BALB/c mice (**Figure 5A**) (25). The TBGR^Tg^ is designed to express ZsGreen reporter proteins from the *Tbx21* gene locus which encodes for T-bet so that ZsGreen expression reflects *Tbx21* transcription (25). Indeed, we identified a substantial population of ZsGreen^+^ cells among thymic *i*NKT cells of TBGR^Tg^ BALB/c mice (**Figure 5A**), and these ZsGreen^+^ *i*NKT cells corresponded to NKT1 cells based on their intracellular PLZF and T-bet protein expression (**Figure 5B**, left). In contrast, TBGR^Tg^ IL-2Rβ^Tg^ BALB/c failed to generate ZsGreen^+^T-bet^+^ *i*NKT cells (**Figure 5B**, right), affirming the detrimental effect of IL-2Rβ^Tg^ on NKT1 cell generation.

Of note, we found in both WT and IL-2Rβ^Tg^ thymocytes a distinct population of *i*NKT cells with intermediate amounts of ZsGreen and T-bet expression, *i.e.* ZsGreen^int^T-bet^int^ (**Figure 5B**, cells gated in blue), whose developmental status was unclear to us. To further characterize these ZsGreen^int^T-bet^int^ *i*NKT cells, we divided TBGR^Tg^ BALB/c *i*NKT cells into 3 populations, *i.e.* I, II, and III, based on their progressive increase in ZsGreen reporter expression. Accordingly, the ZsGreen^int^T-bet^int^ cells corresponded to population II, while ZsGreen^hi^T-bet^hi^ cells were defined as population III (**Figure 5C**). Thymic *i*NKT cells that did not express ZsGreen were referred to as population I. Terminally differentiated NKT1 cells, as identified as population III, expressed large amounts of T-bet and CD44 (**Figure 5C** and **Supplementary Figure 6E**) (11). ZsGreen^int^T-bet^int^ cells (population II), on the other hand, expressed significantly smaller amounts of T-bet and CD44 but showed greater abundance of TCRβ. Importantly, ZsGreen^int^T-bet^int^ cells had not yet induced CD122 expression, suggesting that they are NKT1-lineage committed but not fully differentiated NKT1 cells (**Figures 5C, D** and **Supplementary Figure 6E**) (35). Along these lines, the ZsGreen^int^T-bet^int^ population did not contain RORγt^+^ *i*NKT cells (**Figure 5D**), indicating that NKT17 cell differentiation is branched off before the appearance of ZsGreen^int^T-bet^int^ population II *i*NKT cells. Thus, we identified a new subpopulation of thymic *i*NKT cells, *i.e.* ZsGreen^int^T-bet^int^ *i*NKT cells, that are NKT1-lineage committed but are not fully differentiated into mature NKT1 cells. Because ZsGreen^+^T-bet^+^ *i*NKT cells were missing in IL-2Rβ^Tg^ thymocytes, these results further suggest that the forced and premature expression of IL-2Rβ interferes with the terminal differentiation of T-bet^+^ NKT1 cells but did not inhibit the induction of T-bet expression itself. Collectively, these results unveil an unexpected negative effect of IL-2Rβ on *i*NKT subset differentiation.

### Thymic *i*NKT Cell Differentiation in IL-15-Infused IL-2Rβ^Tg^ BALB/c Mice

**Figure 6:**
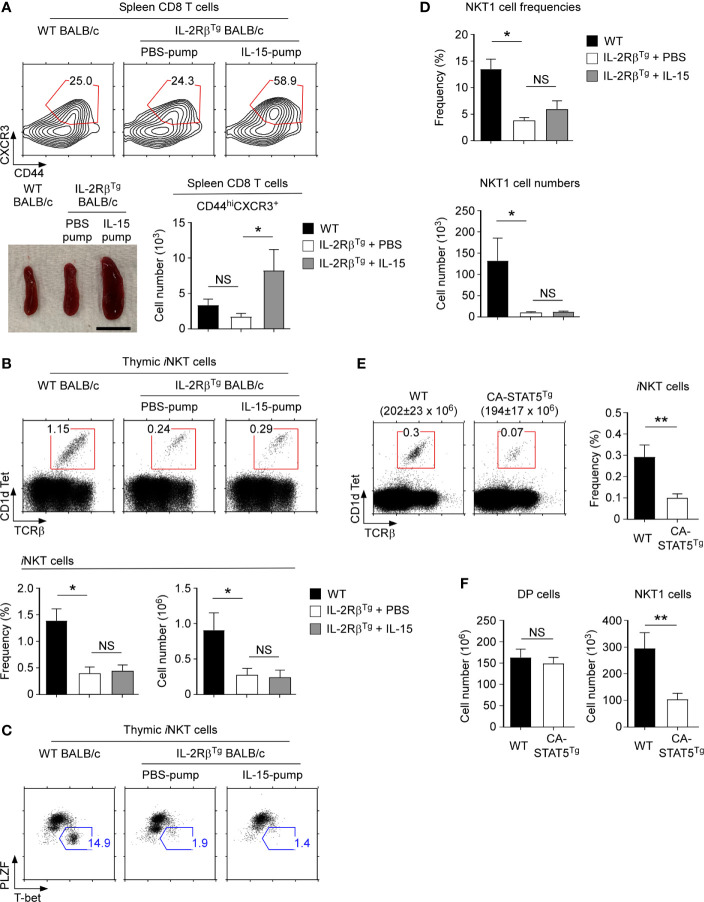
Thymic *i*NKT cell differentiation in IL-15-infused IL-2Rβ^Tg^ BALB/c mice **(A)** Effects of recombinant IL-15- or PBS-releasing Alzet osmotic pump installation on IL-2Rβ^Tg^ BALB/c mice. Spleen CD8 T cells of IL-2Rβ^Tg^ BALB/c mice that were infused with IL-15 or PBS for 10 days were assessed for the accumulation of memory-phenotype CD8 T cells by CD44 *versus* CXCR3 staining. Frequency and numbers of CD44^hi^CXCR3^+^ CD8 T cells are representative of 2 independent experiments with 4 IL-15 pump and 3 PBS pump implanted IL-2Rβ^Tg^ BALB/c mice (top). Picture shows spleens of IL-2Rβ^Tg^ BALB/c mice that were implanted with PBS or IL-15 Alzet pumps (bottom). Scale bar = 1 cm. **(B)** Frequencies and numbers of thymic *i*NKT cells of IL-2Rβ^Tg^ BALB/c mice implanted with IL-15- or PBS-releasing Alzet osmotic pumps. Dot plots are representative (top) and graphs are summary (bottom) of 3 independent experiments with a total of 5 IL-15 pump and 4 PBS pump implanted IL-2Rβ^Tg^ BALB/c mice. **(C)** Thymic *i*NKT subset composition IL-2Rβ^Tg^ BALB/c implanted with IL-15 or PBS-releasing Alzet osmotic pumps. NKT1 cells were identified by PLZF *versus* T-bet of HSA^lo^ mature thymic *i*NKT cells. Results are representative of 3 independent experiments with 5 IL-15 pump and 4 PBS pump implanted IL-2Rβ^Tg^ BALB/c mice. **(D)** Frequencies and numbers of NKT1 cells in IL-2Rβ^Tg^ BALB/c implanted with IL-15 or PBS-releasing Alzet osmotic pumps. Results are summary of 3 independent experiments with 5 IL-15 pump and 4 PBS pump implanted IL-2Rβ^Tg^ BALB/c mice. **(E)** Frequency of thymic *i*NKT cells in CA-STAT5^Tg^ and littermate C57BL/6 mice. The dot plots show thymic *i*NKT cells as identified by CD1dTet *versus* TCRβ staining (left). The total thymocyte numbers are shown above the dot plots as the means ± SEM (left). The bar graph shows the frequency of *i*NKT cells from the indicated mice (right). The dot plot is representative, and the bar graphs are a summary of 4 independent experiments with a total of 7 CA-STAT5^Tg^ and 8 littermate WT mice **(F)** Cell numbers of DP thymocytes and thymic NKT1 cells from CA-STAT5^Tg^ and littermate WT C57BL/6 mice. Bar graph shows the summary from a total of 7 CA-STAT5^Tg^ and 5 littermate WT mice. *P < 0.05; **P < 0.01. NS, Not Significant.

To understand how IL-2Rβ overexpression would affect thymic *i*NKT cell generation, we considered two alternative but not mutually exclusive hypotheses. First, the increased abundance of IL-2Rβ proteins in IL-2Rβ^Tg^ mice could increase the binding and consumption of IL-15, thus diminishing the availability of IL-15 for *i*NKT cell development. IL-15 is critical for the generation of *i*NKT cells in general and specifically for NKT1 cells (11, 36). Consequently, the detrimental effect of IL-2Rβ^Tg^ on *i*NKT cells could have been due to insufficient IL-15 availability, partly phenocopying the effect of IL-15-deficiency (37). In a second scenario, we focused on the timing of IL-2Rβ expression. IL-2Rβ is normally not induced in immature *i*NKT cells but forcibly expressed in IL-2Rβ^Tg^ mice. Premature IL-2Rβ signaling could interfere with *i*NKT cell generation and specifically with NKT1 cell differentiation, albeit the molecular mechanism would be unclear. To discriminate between these possibilities, we implanted IL-2Rβ^Tg^ mice with osmotic pumps that release recombinant IL-15 to supply excess amounts of IL-15 *in vivo* (**Figure 6A**). After 10 days of IL-15 pump installation in IL-2Rβ^Tg^ mice, we found a dramatic increase in spleen size and increased frequencies of NK cells whose maintenance depends on IL-15 (38) (**Figure 6A** and **Supplementary Figure 7A**). Moreover, the frequency of CD8 T cells and specifically the number of CD44^hi^ CXCR3^+^ memory-phenotype CD8 T cells were substantially increased in mice that were implanted with IL-15- but not with PBS-releasing pumps (**Figure 6A** and **Supplementary Figure 7B** **)**. These results were consistent with the effects of increased IL-15 availability *in vivo* (39). The generation of thymic *i*NKT cells in IL-2Rβ^Tg^ BALB/c mice, however, remained unaffected by the IL-15 infusion (**Figure 6B**), and the *i*NKT subset composition also remained unaltered (**Figures 6C, D**). Thus, despite supplemented with excess amounts of IL-15, the IL-2Rβ^Tg^ mice were still impaired in thymic *i*NKT cell differentiation, with both dramatically reduced frequencies and numbers of NKT1 cells (**Figures 6C, D**). Along these lines, the thymic generation of Foxp3^+^ Treg cells which depend on IL-2 and IL-2 receptor signaling (33) was unaffected in IL-2Rβ^Tg^ mice (**Supplementary Figure 7C**), which further supports that the availability of intrathymic cytokines remains unaltered by IL-2Rβ overexpression. Altogether, these results suggested that it is unlikely that increased consumption and reduced availability of *in vivo* IL-15 would account for the lack of NKT1 cells in IL-2Rβ^Tg^ mice.

### Constitutively Active STAT5 Suppresses the Development of Thymic *i*NKT Cells

To gain further mechanistic insights into whether and how premature IL-2Rβ expression would impair *i*NKT cell generation, we considered that STAT5 phosphorylation is a major downstream event of IL-2Rβ signaling (40). The transcription factor STAT5 plays a critical role in T cell development as it induces the expression of prosurvival molecules, such as Bcl-2, and controls the activity of several key transcription factors in T cell differentiation (40, 41). Because immature ST0 *i*NKT cells lack IL-2Rβ (**Figure 1C**), we postulated that ST0 *i*NKT cells of WT mice would not induce phospho-STAT5 (pSTAT5) upon IL-15 stimulation. ST0 *i*NKT cells in IL-2Rβ^Tg^ mice, on the other hand, would have induced substantial amounts of pSTAT5 due the premature expression and signaling of IL-2Rβ.

If it would be the premature STAT5 activation which suppresses *i*NKT cell generation, we hypothesized that a constitutively active STAT5b transgene (CA-STAT5^Tg^) would also impair *i*NKT cell generation (24). Indeed, we found that both the frequency and number of thymic *i*NKT cells were dramatically reduced in CA-STAT5^Tg^ mice compared to those in WT littermate controls (**Figure 6E**). Moreover, assessing the composition of thymic *i*NKT subsets in CA-STAT5^Tg^ mice (**Supplementary Figure 8A**) demonstrated that the generation of NKT1 cells was impaired by ectopic STAT5 activation (**Figure 6F**). T cell development in general, however, was not negatively affected by the constitutive activation of STAT5 (**Supplementary Figure 8B**). As such, the cell numbers of preselection DP thymocytes remained unaffected (**Figure 6F**), and the generation of memory-phenotype CD8 T cells was rather increased in CA-STAT5^Tg^ thymocytes (**Supplementary Figure 8C**). Thus, the detrimental effect of STAT5 activation was limited to the generation of *i*NKT cells. Collectively, these results identify and demonstrate STAT5 as a major player acting downstream of IL-2Rβ to control the development of thymic *i*NKT cells.

## Discussion

*i*NKT cells comprise three major subsets that are characterized by distinct transcription factor and cytokine expression. Although TCR signaling is considered to play a decisive role in the generation and subset differentiation of *i*NKT cells (42, 43), intrathymic cytokines also play important roles in these processes (44). As such, the γc family cytokine IL-15 is a critical factor in thymic *i*NKT cell generation that also contributes to the subset specification of *i*NKT cells (11). Consistent with an IL-15 requirement for NKT1 cell differentiation, the signaling-competent IL-15 receptor heterodimer—*i.e.*, IL-2Rβ/γc—is mostly expressed on NKT1 cells, but not on NKT2 and NKT17 cells (10). This raises the question whether IL-2Rβ expression is specifically induced on NKT1 cells or whether IL-2Rβ could be induced on all *i*NKT cells but is then terminated upon differentiation into NKT2 and NKT17 cells. Furthermore, it was unclear whether IL-2Rβ expression would be sufficient to impose NKT1 subset fate so that forced expression of IL-2Rβ would direct *i*NKT cells into NKT1 cells. Here, we addressed these questions using a series of genetically engineered mouse models, and we identified IL-2Rβ as a critical regulator of thymic *i*NKT cell generation whose abundance and timing of expression dramatically affected the generation of *i*NKT cells. These findings reveal previously unappreciated aspects of cytokine receptor expression in controlling the development of *i*NKT cells, and they propose a model of *i*NKT differentiation that is regulated by the timing and abundance of cytokine receptor expression.

The generation of thymic *i*NKT cells is currently explained by two competing but not mutually exclusive models of differentiation (7, 45). In the conventional or “linear maturation” model, preselection thymocytes that have recombined a functional Vα14-Jα18 TCRα chain are positively selected to become immature ST0 *i*NKT cells. ST0 *i*NKT cells then mature along a developmental pathway defined by CD44 and NK1.1 expression, culminating in end differentiated CD44^+^NK1.1^+^ ST3 *i*NKT effector cells. Under this scenario, CD44^+^NK1.1^–^ ST2 *i*NKT cells, which are IL-2Rβ-negative, would give rise to ST3 *i*NKT cells that are IL-2Rβ-positive. However, the cellular signals that would induce the initial upregulation of IL-2Rβ remain unknown. Notably, such a requirement for IL-2Rβ expression that precedes the IL-15-mediated generation of NKT1 cells is also found in the alternative “lineage differentiation” model (7, 45). While NKT1 cell differentiation depends on IL-15, the IL-2Rβ, which is necessary for IL-15 signaling, is not expressed on immature *i*NKT cells. Thus, IL-2Rβ expression is carefully timed so that preselection thymocytes lack IL-2Rβ but is then specifically induced on NKT1-committed postselection immature *i*NKT cells to drive their maturation into NKT1 subset *i*NKT cells.

The molecular pathway that upregulates IL-2Rβ and drives NKT1 cell fate is not fully understood. However, it likely involves molecules downstream of TCR signaling because differences in TCR signaling strength are acknowledged to drive *i*NKT subset fate decisions (42). In this regard, the transcription factor Egr2 is of particular interest (46), because its relative abundance is associated with *i*NKT subset differentiation (42). Egr2 expression is upregulated by TCR-induced calcium signaling and correlates with the strength of TCR signaling (42, 47). Notably, NKT2 cell generation is thought to be driven by strong TCR signaling whereas NKT1 cell differentiation is proposed to be mediated by weak TCR signaling (42). Incidentally, NKT2 cells contain large amounts of Egr2 whereas NKT1 cells express small amounts of Egr2. Such distinct amounts of Egr2 expression presumably results in the graded expression of PLZF whose abundance correlates with *i*NKT subset specification (48). Thus, the TCR-centric view proposes that distinct amounts of Egr2 would induce different levels of PLZF, which would then determine *i*NKT subset identity. Interestingly, however, Egr2 was also found to bind the IL-2Rβ promoter and thus to induce IL-2Rβ expression (47). Consequently, we hypothesized that Egr2 could induce the expression of IL-2Rβ in immature *i*NKT cells to permit their differentiation into NKT1 cells. According to this scenario, TCR signaling would be required to specify NKT1 fate by upregulating IL-2Rβ expression, and cytokine receptor signaling would be then required to drive NKT1 subset differentiation by IL-15 signaling. Altogether, such model reconciles the two concurrent perspectives of *i*NKT cell development so that TCR and cytokine signals play distinct but nonredundant roles in the subset differentiation of *i*NKT cells.

Because IL-2Rβ plays such a critical role in NKT1 subset commitment, we were surprised to find that the increased abundance of IL-2Rβ in IL-2Rβ^Tg^ mice did not promote but rather suppressed the generation of thymic *i*NKT cells, specifically of the NKT1 subset. A detrimental effect of forced IL-2Rβ expression had been previously reported for NK cells where a hematopoietic lineage-specific IL-2Rβ transgene was found to suppress the generation of NK1.1^+^ cells (49). Whether this NK1.1^+^ population also comprise *i*NKT cells, including stage 3 NKT1 cells, is unclear from that study. If such would be the case, however, these results would further bolster a detrimental effect of IL-2Rβ on *i*NKT cell generation whose molecular basis remains currently unclear to us.

Nonetheless, we considered two important findings regarding the generation of *i*NKT cells in IL-2Rβ^Tg^ mice. First, unlike NKT1 cells, NKT2 and NKT17 cells in WT mice do not express IL-2Rβ (10) Surprisingly, we found that the few NKT2 and NKT17 cells that arise in IL-2Rβ^Tg^ mice also did not express IL-2Rβ proteins. Therefore, the NKT2 and NKT17 cells in IL-2Rβ^Tg^ mice could be either transgene escapees or they could have downregulated the expression of transgenic IL-2Rβ. We favor the latter case because the IL-2Rβ^Tg^ is driven by the human *CD2* mini-cassette, whose activity is downregulated upon positive selection, so that transgene expression substantially diminishes in postselection thymocytes (50). Regardless, we consider the IL-2Rβ^Tg^ model not adequate to assess the effect of forced IL-2Rβ expression on NKT2 and NKT17 cells, simply because the IL-2Rβ^Tg^ fails to be expressed in these *i*NKT subsets. Second, we wish to underscore that the IL-2Rβ^Tg^ is prematurely expressed on immature thymocytes, including the immature ST0 *i*NKT cells. IL-2Rβ is normally absent in the ST0 *i*NKT precursors of WT mice, an observation contrasting the IL-2Rβ expression in IL-2Rβ^Tg^ ST0 *i*NKT cells which are highly abundant in IL-2Rβ. Consequently, ST0 *i*NKT cells in IL-2Rβ^Tg^ mice can be signaled by intrathymic IL-15 whereas WT ST0 *i*NKT cells cannot respond to IL-15. Based on these results, we hypothesize that premature and possibly sustained IL-15 signaling in immature ST0 *i*NKT cells would be detrimental for NKT1 cell generation.

Additionally, we appreciate the need to determine why premature IL-15 signaling in ST0 *i*NKT cells would negatively affect thymic *i*NKT cell generation. IL-15-induced STAT5 phosphorylation is usually considered beneficial for T cells because it induces the expression of anti-apoptotic Bcl-2 and upregulates metabolic activities. Thus, IL-15 signaling should promote and not inhibit *i*NKT cell generation. However, STAT5 activation also suppresses the induction of Bcl6, which is a critical transcription factor for *i*NKT cell generation (51). Bcl-6 is selectively and highly expressed on ST0 *i*NKT cells before they proliferate and differentiate into mature *i*NKT cells. Bcl-6 is also developmentally necessary because Bcl-6-deficiency is associated with impaired thymic *i*NKT cell generation (51). Importantly, IL-2 receptor-induced pSTAT5 potently suppresses Bcl-6 expression (52–54). Therefore, STAT5 activation in ST0 *i*NKT cells could suppress the expression of Bcl-6, which, in turn, could impair the generation of thymic *i*NKT cells. These results lead to a model of *i*NKT cell development where IL-2Rβ expression must be precisely timed so that ST0 *i*NKT cells suppress IL-2Rβ expression to prevent premature IL-15 signaling, but then a subset of post-ST0 *i*NKT cells would rapidly induce IL-2Rβ to initiate IL-15-induced NKT1 lineage differentiation.

Lastly, we wish to point out that the T-bet-ZsGreen reporter mouse revealed a new developmentally intermediate stage in NKT1 cell development that can be identified by intermediate level of T-bet protein expression and T-bet mRNA transcription (as demonstrated by T-bet-ZsGreen reporter expression). Such ZsGreen^int^T-bet^int^ *i*NKT cells were NKT1 lineage committed but not fully differentiated NKT1 cells, as they lacked RORγt expression but had not fully upregulated T-bet protein expression. The ZsGreen^int^T-bet^int^ *i*NKT cells were further marked by their intermediate level of PLZF protein expression which was not downregulated to the amounts found in mature NKT1 cells (48). Thus, ZsGreen^int^T-bet^int^ *i*NKT cells are NKT1 lineage committed but not fully mature NKT1 cells. Because ZsGreen^int^T-bet^int^ *i*NKT cells were present in both WT and IL-2Rβ^Tg^ mice, these data indicate that the forced IL-2Rβ expression did not interfere with NKT1 lineage commitment but rather with the maturation of NKT1 cells.

Collectively, here, we demonstrated the importance of cytokine signaling in NKT1 lineage differentiation by altering the timing and abundance of IL-2Rβ expression, independently of TCR expression or signaling. These results document the significance of cytokines and their receptor expression in determining *i*NKT lineage fate, and further suggest a role for cytokines in parallel or in association with TCR signaling to shape the effector function of T cells.

## Data Availability Statement

The original contributions presented in the study are included in the article/**Supplementary Material**. Further inquiries can be directed to the corresponding author.

## Ethics Statement

The animal study was reviewed and approved by National Cancer Institute Animal Care and Use Committee.

## Author Contributions

HW designed and performed the experiments, analyzed the data, and contributed to the writing of the manuscript. HK, AC, PA, and RG provided expertise, analyzed the data, and commented on the manuscript. J-HP conceived the project, analyzed the data, and wrote the manuscript. All authors contributed to the article and approved the submitted version.

## Conflict of Interest

The authors declare that the research was conducted in the absence of any commercial or financial relationships that could be construed as a potential conflict of interest.
