# Supplementary material for: The Timing and Abundance of IL-2Rβ (CD122) Expression Control Thymic iNKT Cell Generation and NKT1 Subset Differentiation
Source: Front Immunol. 2021 May 14;12:642856. doi: 10.3389/fimmu.2021.642856 (PMC8161506; doi:10.3389/fimmu.2021.642856)
Supplement: Supplementary file 1 [file DataSheet_1.pdf]

Supplementary Figure 1

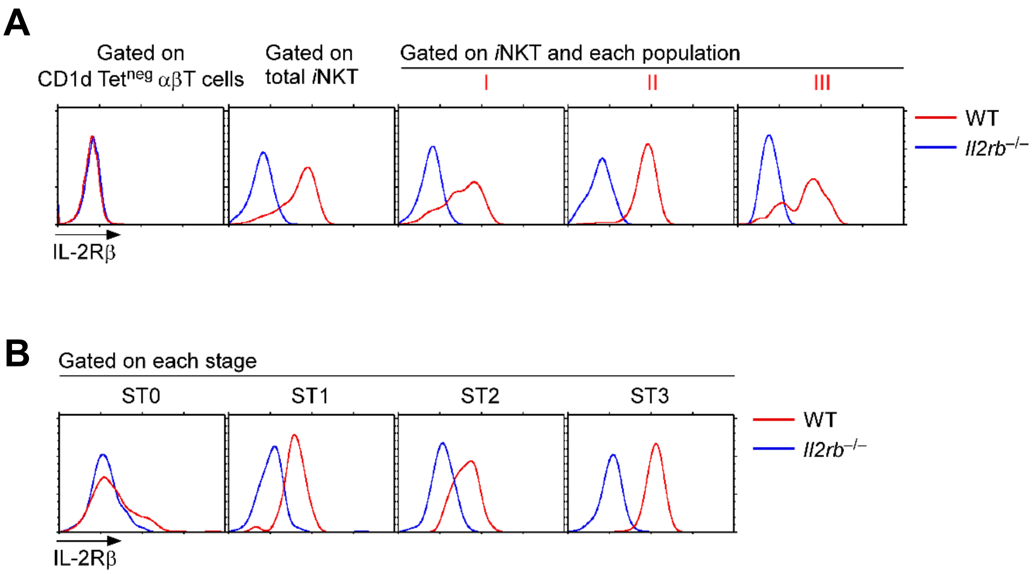

Supplementary Figure 1. IL-2Rβ expression in thymocytes of *Il2rb*$^{-/-}$ and WT littermate control mice

Based on CD69 and CCR7 staining, total thymocytes of *Il2rb*$^{-/-}$ and WT littermate control mice were placed into 5 distinct developmental stages, *i.e.* I – V. Surface IL-2Rβ expression was then assessed on **(A)** thymic *i*NKT cells, and **(B)** ST0 – ST3 thymic *i*NKT cells of WT and *Il2rb*$^{-/-}$ mice to confirm the antigen specificity of anti-IL-2Rβ antibody staining.

Supplementary Figure 2

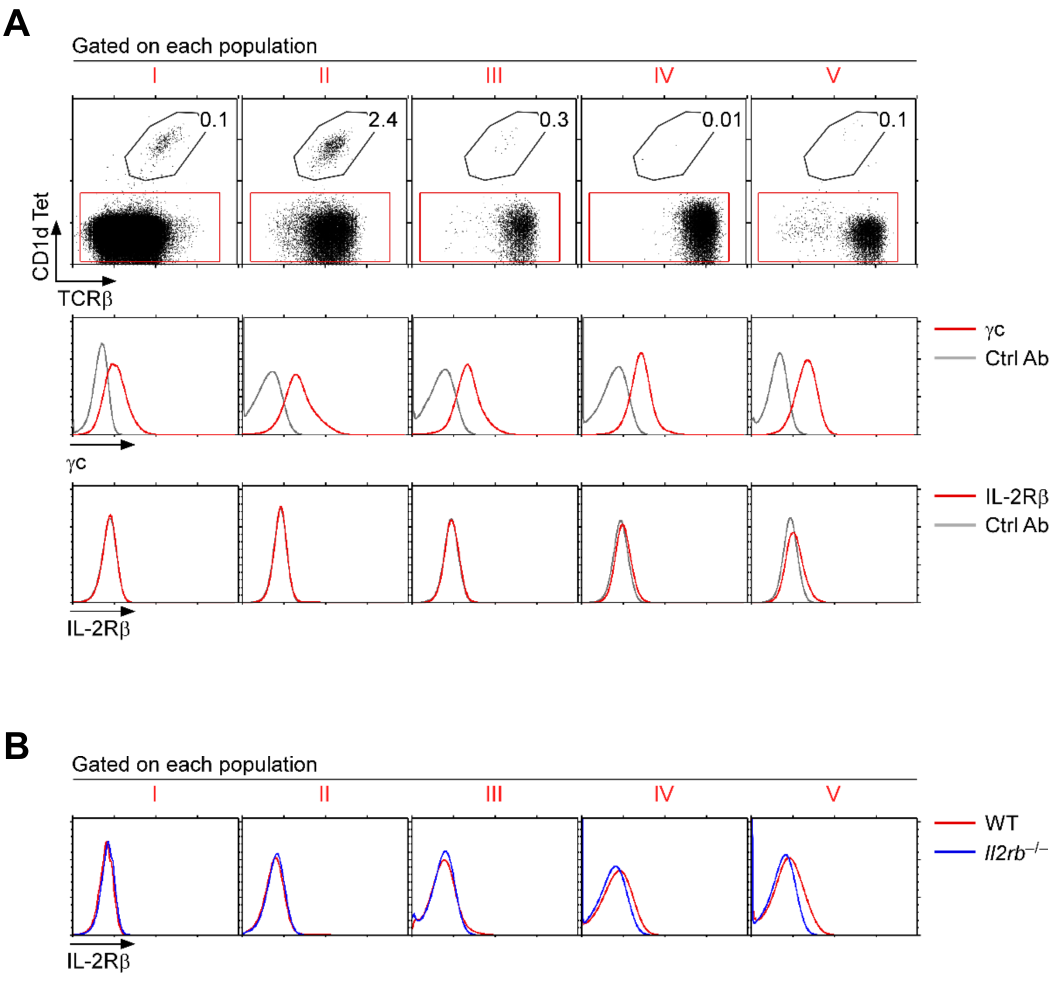

Supplementary Figure 2. Cytokine receptor expression in conventional αβ TCR thymocytes

Based on CD69 and CCR7 staining, total thymocytes of C57BL/6 mice were categorized into 5 distinct developmental stages, *i.e.* I – V.

A. Conventional thymocytes were identified as CD1dTet-negative cells (top, red boxes), and assessed for surface γc (middle) and IL-2Rβ (bottom) cytokine receptor expression. Results are representative of 3 independent experiments.

B. Surface IL-2Rβ expression on stage I – V conventional thymocytes of WT and *Il2rb*$^{-/-}$ mice confirms the antigen specificity of anti-IL-2Rβ antibody staining.

Supplementary Figure 3

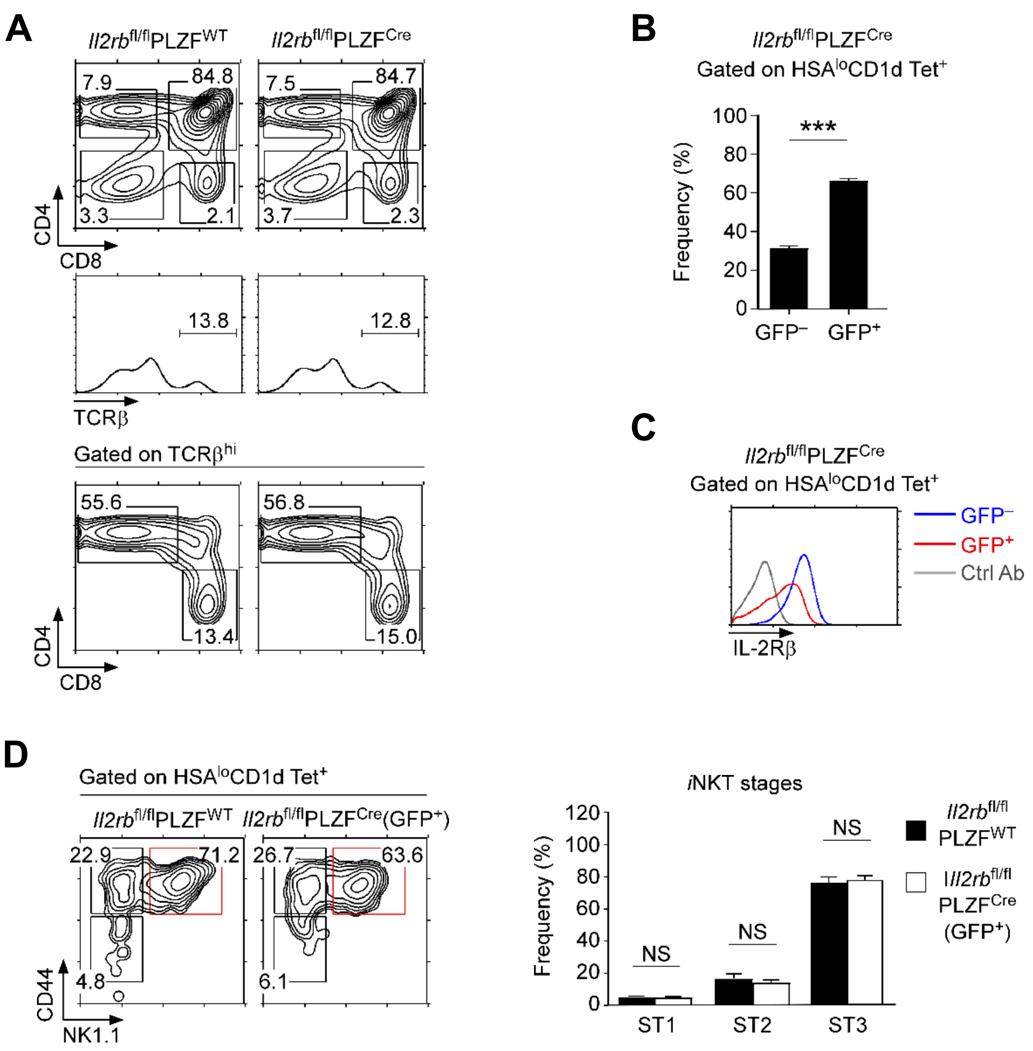

Supplementary Figure 3. Thymocyte and *i*NKT cell development in *Il2rb*$^{fl/fl}$PLZF^{Cre} mice

A. Thymocyte development in *Il2rb*$^{fl/fl}$PLZF^{Cre} mice. The contour plots show the CD4 versus CD8 profile of total thymocytes (top) or TCRb^{hi}-gated (middle) mature thymocytes (bottom) of the indicated mice. The total thymocyte numbers are shown above the contour plots as the means ± SEM.

B. Frequency of GFP^{+} and GFP-negative cells among HSA^{lo}CD1dTet^{+} *i*NKT cells in *Il2rb*$^{fl/fl}$PLZF^{Cre} mice. Results are the summary of 5 independent experiments.

C. IL-2Rβ expression on GFP^{+} and GFP-negative cells among HSA^{lo}CD1dTet^{+} *i*NKT cells of *Il2rb*$^{fl/fl}$PLZF^{Cre} mice. Histogram is representative of 6 analyses.

D. *i*NKT cells were stained for CD44 and NK1.1 to identify ST1, ST2, and ST3 cells as shown in the contour plots (left). The bar graph shows the frequencies of ST1, ST2, and ST3 cells in *Il2rb*$^{fl/fl}$PLZF^{Cre} and *Il2rb*$^{fl/fl}$ littermate mice (right). The data are a summary of 4 independent experiments with a total of 11 *Il2rb*$^{fl/fl}$PLZF^{Cre} and 5 *Il2rb*$^{fl/fl}$ littermate control mice.

Supplementary Figure 4

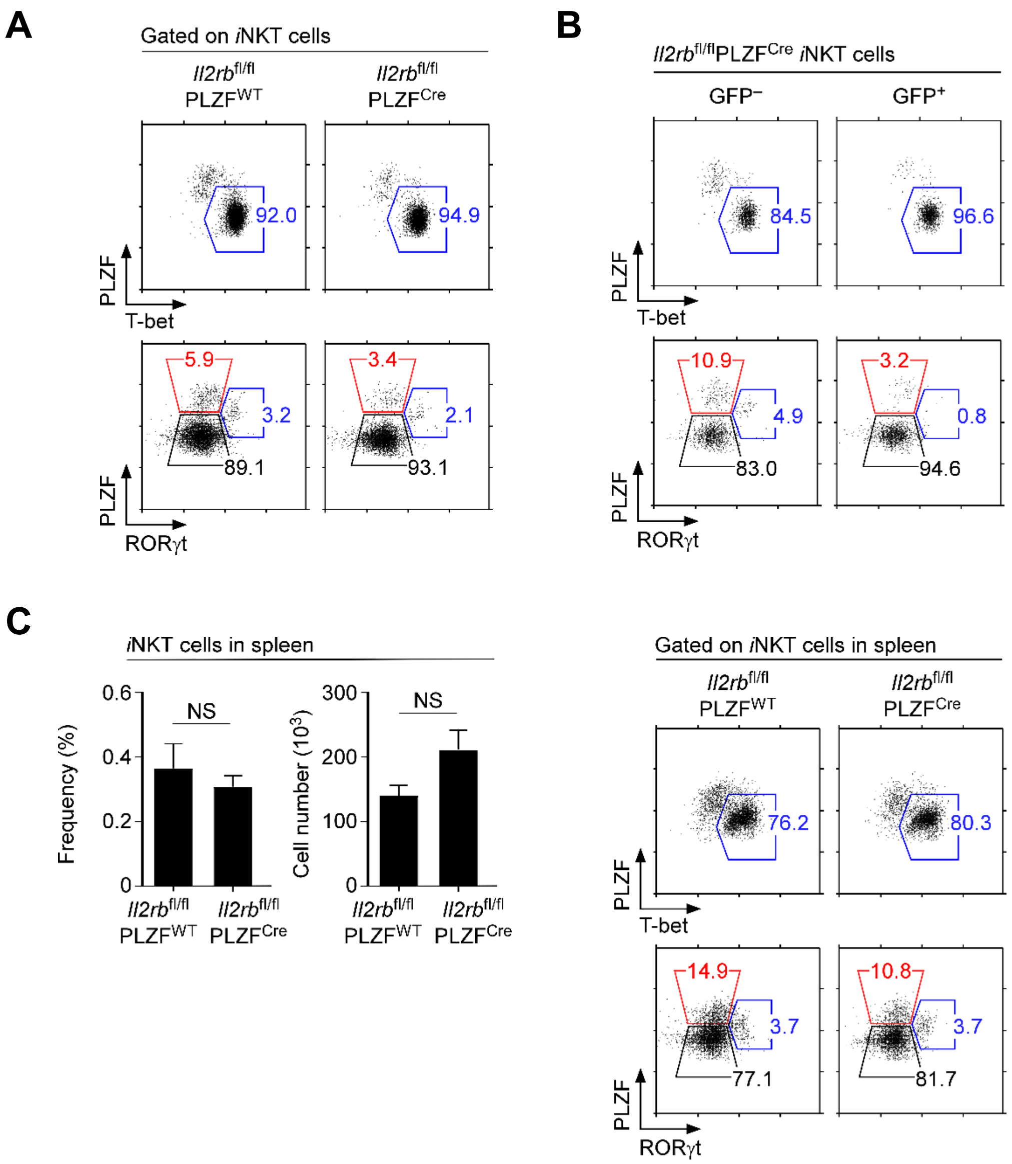

Supplementary Figure 4. *i*NKT cell subset composition in *Il2rb*$^{fl/fl}$PLZF^{Cre} mice

A. The subset composition of thymic *i*NKT cells in *Il2rb*$^{fl/fl}$PLZF^{Cre} and *Il2rb*$^{fl/fl}$ littermate mice were assessed by PLZF versus T-bet (top) and PLZF versus RORγt staining (bottom). Results are representative of 2 independent experiments with a total of **7** a *Il2rb*$^{fl/fl}$PLZF^{Cre} and **3** *Il2rb*$^{fl/fl}$ littermate mice

B. The subset composition of thymic *i*NKT cells in GFP-negative (GFP^{-}) and GFP^{+} *i*NKT cells of *Il2rb*$^{fl/fl}$PLZF^{Cre} mice were assessed by PLZF versus T-bet (top) and PLZF versus RORγt staining (bottom). Results are representative of 2 independent experiments with a total of 7 a *Il2rb*$^{fl/fl}$PLZF^{Cre} and 3 *Il2rb*$^{fl/fl}$ littermate mice

C. Frequency and number of *i*NKT cells in the spleen of *Il2rb*$^{fl/fl}$PLZF^{Cre} and *Il2rb*$^{fl/fl}$PLZF^{WT} mice (left). The subset composition of thymic *i*NKT cells in *Il2rb*$^{fl/fl}$PLZF^{Cre} and *Il2rb*$^{fl/fl}$ littermate mice (right) were assessed by PLZF versus T-bet (top) and PLZF versus RORγt staining (bottom). Results are representative of 3 independent experiments with a total of 8 a *Il2rb*$^{fl/fl}$PLZF^{Cre} and 4 *Il2rb*$^{fl/fl}$ littermate mice.

Supplementary Figure 5

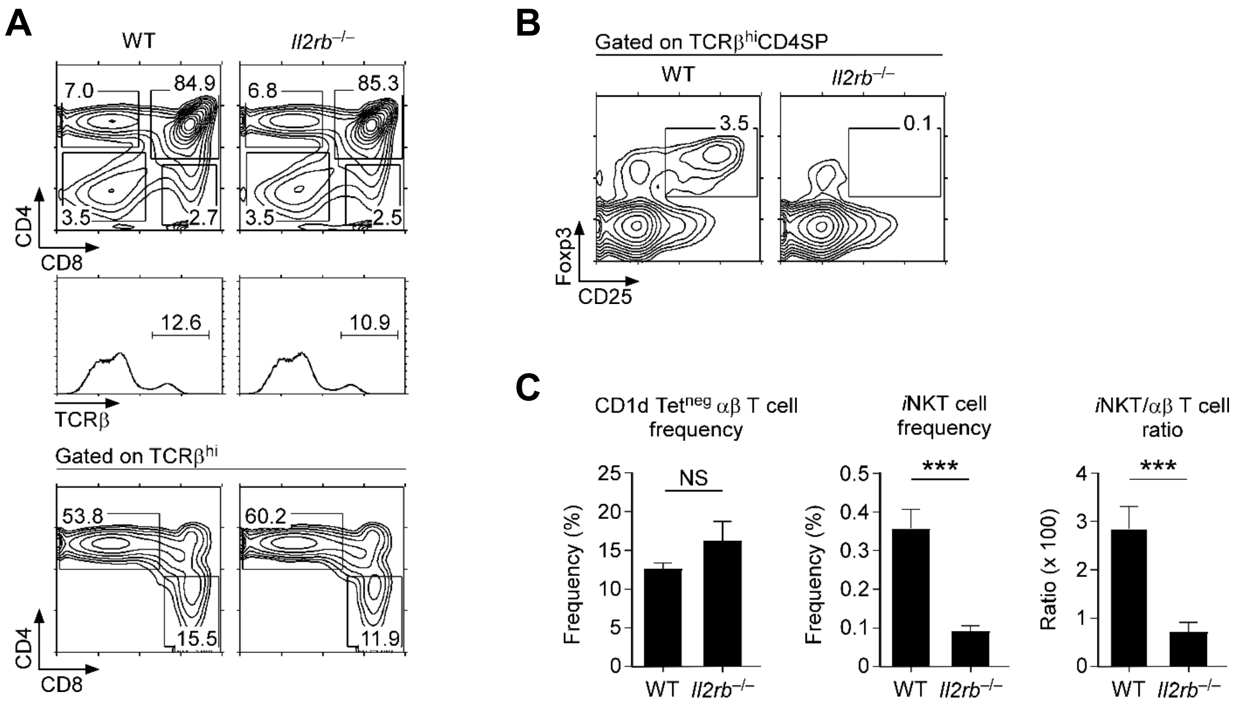

Supplementary Figure 5. Thymic T cell development in IL-2Rβ-deficient mice
A. CD4 versus CD8 profiles of total (top) and TCRβ^{hi}-gated (middle) mature thymocytes (bottom) of *Il2rb*$^{-/-}$ and WT littermate control mice. Results are representative of 2 independent experiments with a total of 4 *Il2rb*$^{-/-}$ and 3 WT littermate control mice.
B. CD25^{+}Foxp3^{+} Treg cell frequencies in CD4SP thymocytes of *Il2rb*$^{-/-}$ and WT littermate control mice. Data are representative of 2 independent experiments with a total of 6 *Il2rb*$^{-/-}$ and 5 WT littermate control mice.
C. Frequencies and ratio of conventional (CD1dTet-negative) αβ T cells and *i*NKT cells in the thymus of *Il2rb*$^{-/-}$ and WT littermate control mice. Frequencies are shown among total thymocytes, and ratios were determined by dividing *i*NKT cell frequencies by αβ T cell frequencies, then multiplied by 100. Results are summary of 3 independent experiments with 10 *Il2rb*$^{-/-}$ and 7 WT littermate control mice.

Supplementary Figure 6

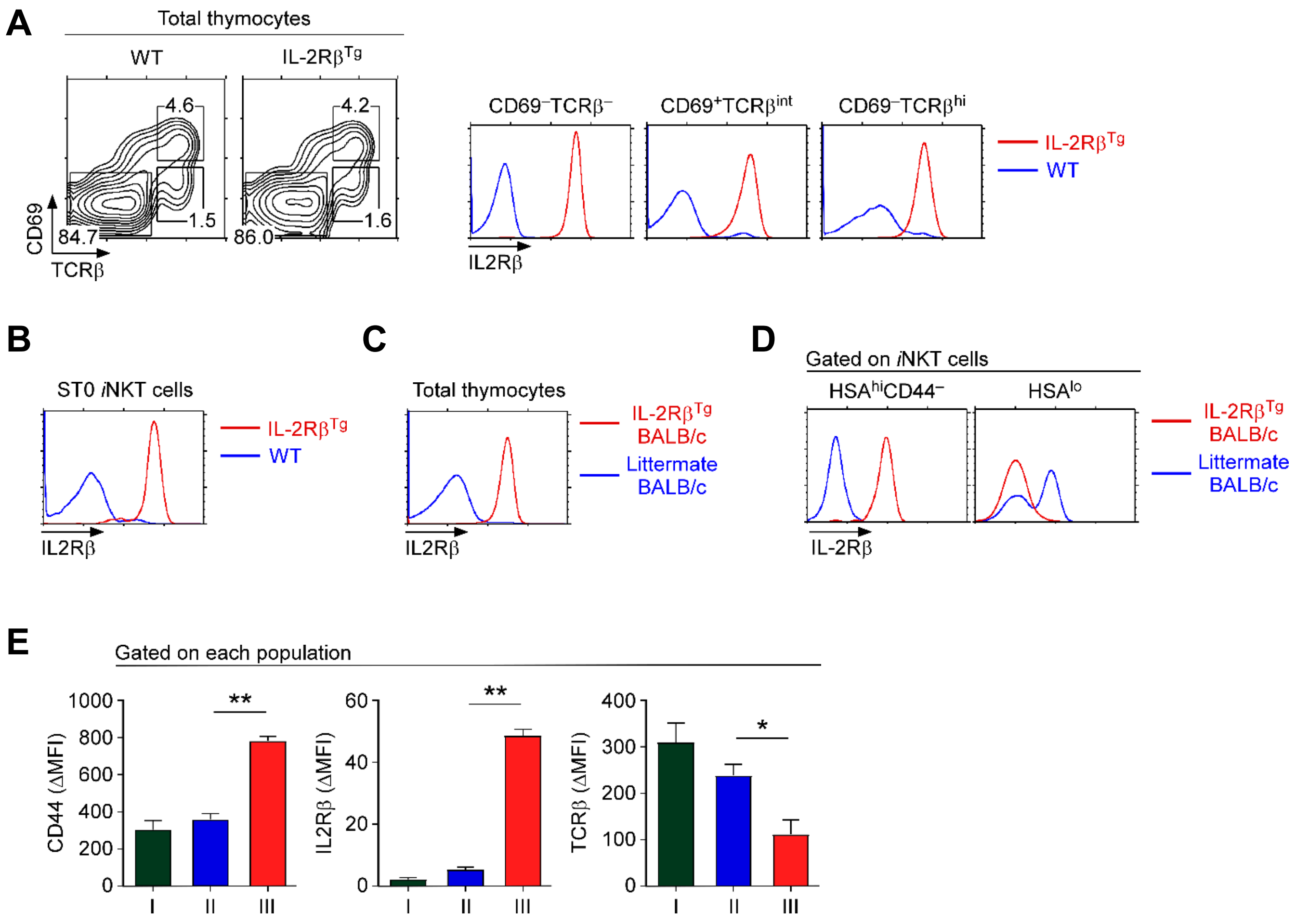

Supplementary Figure 6. IL-2Rβ expression on thymocyte subpopulations

A. Contour plots show CD69 versus TCRβ profiles of IL-2Rβ^{Tg} and WT littermate control thymocytes. Histograms show surface IL-2Rβ expression of the indicated thymocyte subsets in IL-2Rβ^{Tg} and WT littermate control mice. CD69^{-}TCRβ^{-} correspond to preselection thymocytes, CD69^{+}TCRβ^{int} correspond to thymocytes undergoing positive selection, and CD69^{-}TCRβ^{hi} are postselection thymocytes.

B. IL-2Rβ expression on ST0 *i*NKT cells of IL-2Rβ^{Tg} and WT C57BL/6 thymocytes.

C. IL-2Rβ expression on total thymocytes of WT and in IL-2Rβ^{Tg} and WT littermate control BALB/c mice. Results are representative of 3 independent experiments with a total of 6 IL-2Rβ^{Tg} and 5 WT littermate control mice.

D. Surface IL-2Rβ expression on HSA^{hi}CD44^{-} immature and HSA^{lo} mature thymic *i*NKT cells of IL-2Rβ^{Tg} BALB/c and littermate WT mice. The histograms are representative of 2 independent experiments with a total of 3 IL-2Rβ^{Tg} and 3 littermate WT mice.

E. Surface marker expression in thymic *i*NKT sub-populations as identified by T-bet-Zs-Green reporter expression. CD44, IL-2Rβ and TCRβ expression was assessed on population I (T-bet-ZsGreen negative), II (T-bet-ZsGreen intermediate), and III (T-bet-ZsGreen high). Results show the summary of two independent experiments with a total of 6 TBGR^{Tg} BALB/c mice

Supplementary Figure 7

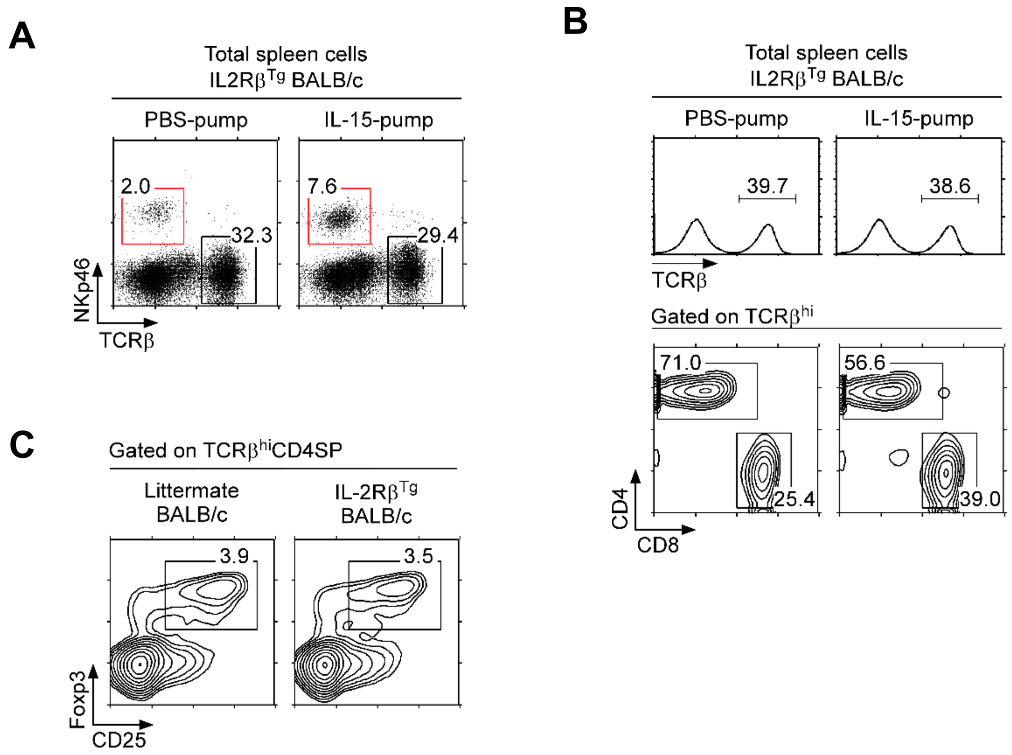

Supplementary Figure 7. Effect of IL-15 osmotic pump implantation on IL-2Rβ^{Tg} mice

A. Spleen NK cells were identified using the NK cell-specific marker NKp46 and by the lack of TCRβ expression. NK cell frequencies were determined in IL-2Rβ^{Tg} mice that were implanted with osmotic pumps releasing recombinant IL-15 or vehicle (PBS) control. Results are representative of 2 independent experiments.

B. Splenocyte analysis of IL-15 osmotic pump installed IL-2Rβ^{Tg} mice. Contour plots (bottom) show the CD4 versus CD8 profile of TCRβ^{+}-gated spleen cells (top) from IL-2Rβ^{Tg} mice that were installed with either PBS- or IL-15-osmotic pumps.

C. CD25^{+} Foxp3^{+} Treg cells in mature CD4SP thymocytes of IL-2Rβ^{Tg} and WT BALB/c mice. Results are representative of 2 independent experiments with a total of 5 IL-2Rβ^{Tg} and 4 WT BALB/c mice.

Supplementary Figure 8

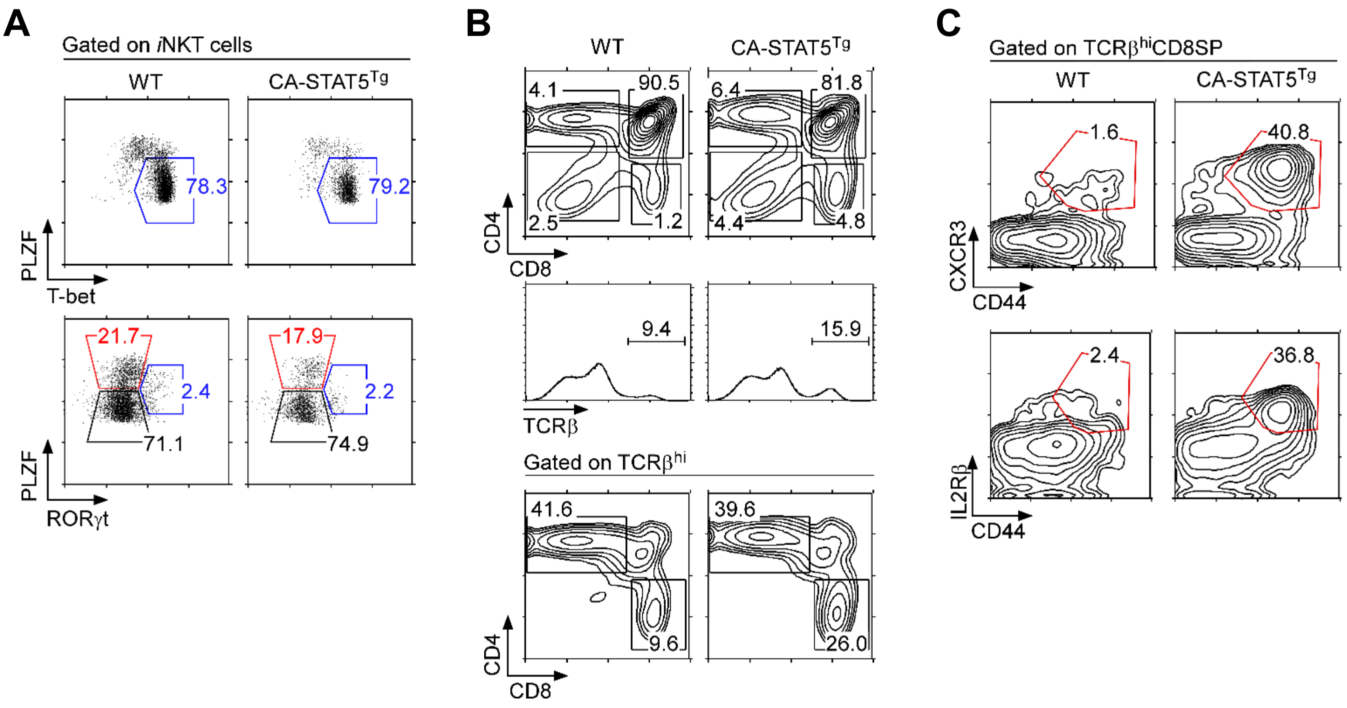

Supplementary Figure 8. T cell development in CA-STAT5^{Tg} mice

A. Thymic *i*NKT subsets in WT and CA-STAT5^{Tg} mice. Results are representative of 2 independent experiments with 3 CA-STAT5^{Tg} and 5 littermate WT C57BL/6 mice.

B. Thymocyte differentiation in CA-STAT5^{Tg} mice. The contour plots show the CD4 versus CD8 profile of total thymocytes (top) or TCRβ^{hi}-gated (middle) mature thymocytes of the indicated mice (bottom). Data are representative of 4 independent experiments with total 8 WT and 7 CA-STAT5^{Tg} mice.

C. Frequencies of memory-phenotype CD8 T cells among CD8SP thymocytes of WT and CA-STAT5^{Tg} mice as identified by CXCR3 versus CD44 (top) and IL-2Rβ versus CD44 expression (bottom). Data are representative of 3 independent experiments with total 7 WT and 6 CA-STAT5^{Tg} mice.
